# Effect of physical exercise on white matter microstructure in chemotherapy-treated breast cancer patients: a randomized controlled trial (PAM study)

**DOI:** 10.1007/s11682-024-00965-9

**Published:** 2025-01-13

**Authors:** Emmie W. Koevoets, Sanne B. Schagen, Anne M. May, Mirjam I. Geerlings, Lenja Witlox, Elsken van der Wall, Martijn M. Stuiver, Gabe S. Sonke, Miranda J. Velthuis, Jan J. Jobsen, Job van der Palen, Michiel B. de Ruiter, Evelyn M. Monninkhof

**Affiliations:** 1 Julius Center for Health Sciences and Primary Care, University Medical Center Utrecht, Utrecht University, Utrecht, The Netherlands; 2 Division of Psychosocial Research and Epidemiology, Netherlands Cancer Institute, Amsterdam, The Netherlands; 3 Brain and Cognition Group, University of Amsterdam, Amsterdam, The Netherlands; 4 Department of General Practice, Amsterdam UMC, Location University of Amsterdam, Amsterdam, The Netherlands; 5 Amsterdam Public Health, Aging & Later Life, and Personalized Medicine, Amsterdam UMC, Location University of Amsterdam, Amsterdam, The Netherlands; 6 Amsterdam Neuroscience, Neurodegeneration, and Mood, Anxiety, Psychosis, Stress, and Sleep, Amsterdam UMC, Location University of Amsterdam, Amsterdam, The Netherlands; 7 Department of Medical Oncology, University Medical Center Utrecht, Utrecht University, Utrecht, The Netherlands; 8 Center for Quality of Life, Netherlands Cancer Institute, Amsterdam, The Netherlands; 9 Department of Medical Oncology, Netherlands Cancer Institute, Amsterdam, the Netherlands; 10 Netherlands Comprehensive Cancer Organisation (IKNL), Utrecht, The Netherlands; 11 Medical School Twente, Medisch Spectrum Twente, Enschede, The Netherlands; 12 Section Cognition, Data and Education, Universiteit Twente, Enschede, The Netherlands

**Keywords:** Exercise, Breast cancer, White matter, Fatigue, Cognition

## Abstract

Physical exercise is a promising intervention to improve brain white matter integrity. In the PAM study, exercise intervention effects on white matter integrity were investigated in breast cancer patients. Chemotherapy-treated breast cancer patients with cognitive problems were randomized 2–4 years post-diagnosis to an exercise (*n* = 91) or control group (*n* = 90). The 6-month exercise intervention consisted of four hours/week of aerobic and resistance training. White matter integrity was measured at baseline and 6-month follow-up with fractional anisotropy (FA) and mean diffusivity (MD), which were derived from magnetic resonance diffusion tensor imaging (DTI). Both DTI metrics were analyzed whole brain and voxel-wise with a modified tract based spatial statistics (TBSS) procedure. Other measurements included cognition and physical fitness. Exercise effects were analyzed with multiple regression analyses. An explorative analysis was conducted in highly fatigued patients. DTI scans were available for 69 patients of the intervention (age = 52.3 ± 8.9yrs.) and 72 patients of the control group (age = 53.2 ± 8.6yrs.). Whole brain and voxel-wise analyses revealed no significant intervention effects on FA and MD. In highly fatigued patients (exercise: *n* = 32; control: *n* = 24), significant clusters of decreased FA post-intervention were observed in the left inferior and superior longitudinal fasciculus. Mean FA in these clusters was not predictive of cognition. A 6-month exercise intervention did not affect white matter integrity in chemotherapy-treated breast cancer patients. However, in highly fatigued breast cancer patients a significant FA decrease was observed post-intervention. The direction of these results is unexpected, and more research is needed to further understand these results.

**Supplementary Information:**

The online version contains supplementary material available at 10.1007/s11682-024-00965-9.

## Introduction

Approximately 25% of all breast cancer patients show cognitive decline after diagnosis and treatment, especially after chemotherapy (Dijkshoorn et al., 2021). Multiple cognitive domains are affected, including learning and memory, attention, and executive functioning (Wefel et al., 2015). The severity of cancer-related cognitive impairment is reported to be of a mild to moderate nature but can have a significant impact on daily activities and quality of life (Ahles & Root, 2018; Lange et al., 2019; Wefel et al., 2015). Successful interventions targeting the underlying mechanism of cognitive functioning are needed and physical exercise shows promise (Campbell et al., 2020; Wefel et al., 2015).

Brain white matter, which is essential for cognition, seems vulnerable to the neurotoxic effects of chemotherapeutic agents (Matsos et al., 2017). Both cross-sectional and longitudinal studies have observed impaired white matter microstructure in chemotherapy-treated cancer patients (Amidi & Wu, 2019). Even years after chemotherapy treatment, some studies have reported impaired white matter microstructural integrity in cancer patients compared to controls (De Ruiter et al., 2012; Stouten-Kemperman et al., 2015). Additionally, several (prospective) studies in cancer patients showed that worse white matter microstructural integrity is associated with lower cognitive performance (Abraham et al., 2008; Chen et al., 2024; Daniel et al., 2023; Deprez et al., 2011; Deprez et al., 2012; McDonald, 2021Sabin et al., 2018).

In white matter tracts, diffusion of water molecules is often measured with magnetic resonance diffusion tensor imaging (DTI). Fractional anisotropy (FA) and mean diffusivity (MD) are two frequently used DTI parameters. FA characterizes the directionality of diffusion of water molecules, with values ranging from 0 (free diffusion of water molecules) to 1 (complete restriction of water molecules in one direction). In white matter tracts, diffusion of water molecules is highly restricted due to the presence of myelin. FA values are, therefore, particularly high within these tracts. The DTI metric MD characterizes the overall magnitude of diffusion, and MD is generally lower (more restricted) in brain tissue (white and gray matter) than in cerebrospinal fluid. Many studies have demonstrated that DTI is suitable to measure changes in white matter integrity.

Exercise is a promising non-pharmacological intervention to improve cognition, potentially by enhancing white matter integrity. Several studies performed in healthy (older adult) and patient populations reported a positive association between (regional) FA and cardiovascular fitness measures (Fissler et al., 2017; Hayes et al., 2015; Johnson et al., 2012; Liu et al., 2012; Opel et al., 2019; Perea et al., 2016; Tseng et al., 2013). The relationship between physical activity levels and white matter integrity is a topic that has been explored in other research areas, including aging and psychiatry. The results are somewhat inconclusive, with a tendency towards a positive association. A six-month exercise intervention in patients with schizophrenia resulted in a significant increase in white matter integrity (Svatkova et al., 2015). Similarly, a one-year aerobic exercise intervention in patients with amnestic mild cognitive impairment demonstrated that individual gains in physical fitness were associated with improved regional white matter integrity (Tarumi et al., 2020). There is a paucity of published research examining the impact of exercise and fitness on neural outcomes in breast cancer survivors. However, a recent study by Lesnovskaya et al. (Lesnovskaya et al., 2023) identified a positive correlation between aerobic fitness (as measured by a submaximal test) and resting-state functional connectivity. Additionally, a small pilot study (*n* = 10) suggests that a 12-week aerobic exercise intervention may influence functional brain network organization and cognition in breast cancer survivors (Page et al., 2024).

Still, studies examining exercise intervention effects on white matter integrity remain sparse, and no studies have been performed in chemotherapy-treated cancer patients.

In the randomized Physical Activity and Memory (PAM) study, we investigated the effects of a 6-month exercise intervention on white matter integrity in cognitively affected (self-reported and confirmed by tests) chemotherapy-treated breast cancer patients, who were 2–4 years after diagnosis. To gain further insight into the potential association between white matter integrity and cognitive performance, we analyzed this relationship across the entire sample. Additionally, we examined the effects of the exercise intervention in a subgroup of highly fatigued patients. Previously, we reported that physical exercise had no positive impact on tested cognitive performance. However, we did observe beneficial effects on subjective cognitive functioning, fatigue, depression, and quality of life (Koevoets et al., 2022). In a subgroup of highly fatigued patients, we did find improvements in tested cognitive performance, in the domains of processing speed, and learning and memory.

## Methods

### Study design

The design of the PAM study and results regarding cognitive functioning, patient-reported outcomes, and physical fitness, have been published elsewhere (Koevoets et al., 2022; Witlox et al., 2019). In short, the PAM study was a multi-center randomized controlled trial with two study arms (exercise group versus control group). Patients were recruited between December 2016 and September 2020. Measurements were performed before randomization and after six months at the University Medical Center Utrecht (UMCU), The Netherlands. The study was approved by the Medical Ethics Committee of the UMCU, and all patients provided written informed consent before baseline measurements. This trial was registered in the Netherlands Trial Registry: Trial NL5924 (NTR6104), https://www.trialregister.nl/trial/5924.

Women were eligible for inclusion if they were 2–4 years after stage I-III breast cancer diagnosis, had been treated with (neo)adjuvant chemotherapy, were 30–75 years old, had no evidence of disease recurrence, and reported to be less active than recommended (≤ 150 min of moderate-to-vigorous physical activity per week). Additionally, patients reported cognitive problems and showed lower-than-expected performance on online neuropsychological tests (see 2.2). Patients were ineligible for study inclusion if they could not safely complete the exercise program, could/would not undergo MRI scanning, had a known neurological disorder affecting cognition (e.g. dementia, multiple sclerosis), and/or were planning to stop or switch endocrine therapy four months before or during the study period.

### Recruitment and randomization

**Fig. 1:**
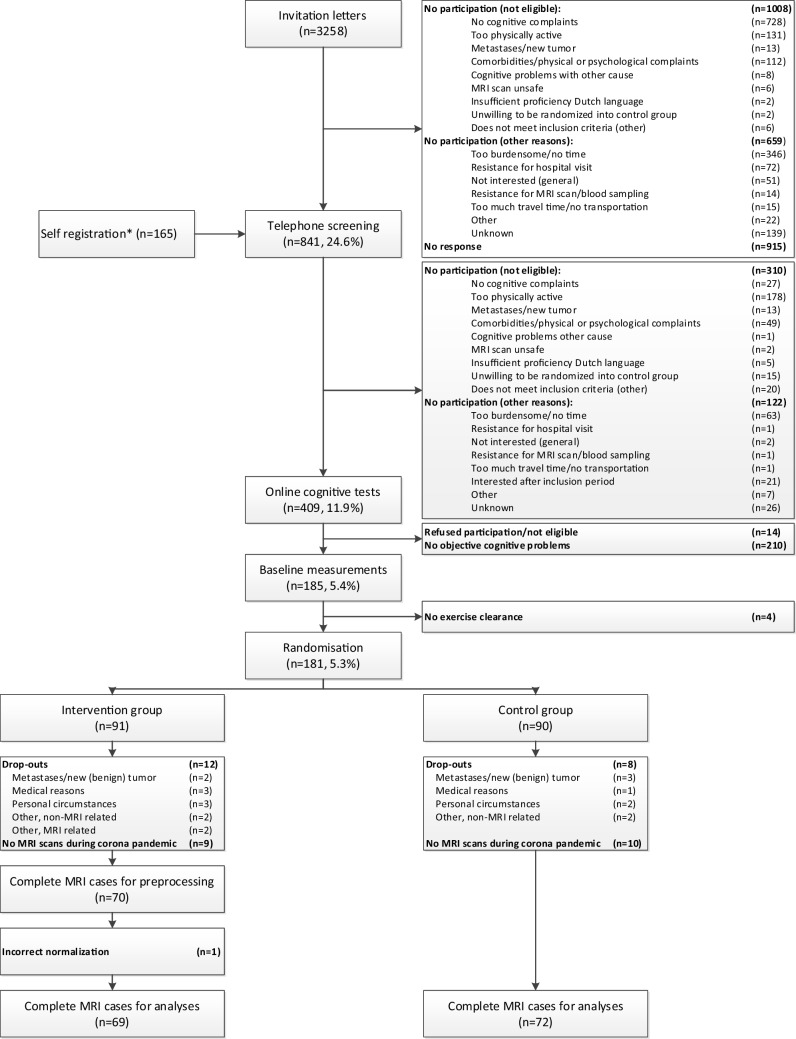
Flowchart of inclusion and randomization procedures of the Physical Activity and Memory (PAM) study patients. *Information through social media, pamphlets, and by word of mouth. **During the last months of the PAM study, a lockdown during the COVID-19 pandemic impacted study procedures. Patients were unable to visit the hospital for research purposes, resulting in missing MRI scans (*n* = 19) and cardiopulmonary exercise tests (*n* = 13). Additionally, we had to modify the exercise program to be home-based (*n* = 7), providing them with exercise materials such as dumbbells and a stationary bike

Most patients were invited through invitation letters from their treating physicians (*n* = 3,258) (Fig. 1). Following telephone screening for eligibility (including a semi-structured interview about cognitive complaints and activity level) (*n* = 841), 409 patients were requested to complete an online neuropsychological test battery, the Amsterdam Cognition Scan (ACS) (Feenstra et al., 2018). The ACS was used to confirm below-expected performance (≥ 1 normative standard deviation below average performance, controlled for sex and age) on at least two cognitive tests in separate cognitive domains of the total 11 outcomes from five different cognitive domains (Supplementary Table 1). Subsequently, eligible patients were invited for baseline measurements at the UMCU. Afterwards, patients were randomized to the exercise or control group (1:1) using a computer-generated sequence, to ensure blinded treatment allocation, provided by UMCU data-management. Randomization was stratified by age category (30–44, 45–59, 60–75) and endocrine therapy use (yes, no).

### Exercise intervention and control group

The 6-month exercise intervention consisted of two hours/week of aerobic (50%) and strength training (50%) delivered by a physiotherapist close to the patient’s home, and two hours/week of Nordic or power walking. The initial intensity of the exercise program was based on physical fitness at baseline (cardiopulmonary exercise test; CPET), possible constraints, and repetition maximum tests. The aerobic intensity and resistance exercises during the supervised sessions were progressively increased as the program advanced (Supplementary Table 2). The intensity of the Nordic or power walking sessions was set at 55%−65% of the heart rate reserve, as it is challenging to achieve higher values with this activity. Heart rate monitors provided by the study team were used to measure intensity. Approximately one month after the start of the intervention, a member of the study team visited the supervised training location to verify protocol adherence.

Patients who were randomized to the control group were requested to continue with their habitual physical activities. After follow-up measurements, a supervised 12-week exercise program was offered to control patients.

### MRI acquisition and preprocessing

The complete MRI protocol has been described elsewhere (Witlox et al., 2019). MRI data were acquired on a Philips 3.0 Tesla MRI scanner at the UMCU using a standardized protocol (acquisition time 25 min). The DTI sequence (TR/TE = shortest/90, 40 directions, b-value 1500, 56 slices, 2.50 mm isotropic voxels) was used for assessing changes in white matter microstructure. The sagittal 3D T1-weighted sequence (TR/TE = 7.9/4.5 ms; 192 slices; 1.00 mm isotropic voxels) was used for registration purposes and anatomical reference. All scans were visually inspected by a radiologist of the UMCU for incidental findings.

For preprocessing of the DTI data, we used a modified tract based spatial statistics (TBSS) procedure that is more sensitive to changes in white matter microstructure than conventional TBSS (Supplementary material (text)) (De Ruiter et al., 2021; Mzayek et al., 2021; Schwarz et al., 2014; Smith et al., 2006). This procedure utilizes selected routines from the FMRIB Software Library (FSL6.0, Oxford University, UK; http://www.fmrib.ox.ac.uk/fsl) (Smith et al., 2004) and the Advanced Normalization Tools (ANTs) (Avants et al., 2011). Each step was visually inspected for artefacts. FA and MD images were smoothed with a 6 mm full width at half-maximum (FWHM) Gaussian kernel. A binary mask was constructed to analyze voxels with a mean FA value of > 0.2, restricting the analyses mainly to white matter.

### Other measures

#### Sociodemographic data

Age, education, menopausal status, and age at menopause were assessed by a questionnaire. Clinical characteristics were obtained from medical records, and medication use, including endocrine therapy, was collected during interviews.

#### Cognition

We used the Hopkins Verbal Learning Test-Revised total recall score (HVLT-R total recall) to measure memory functioning, which was the primary outcome measure of the PAM study. This is a recommended test for memory functioning in cancer patients (Benedict et al., 1998; Wefel et al., 2011). Additionally, the Reaction Time task of the ACS was used to measure processing speed, since this measure showed significant intervention effects in highly fatigued patients (Koevoets et al., 2022) and processing speed is associated with white matter integrity (e.g. (Burzynska et al., 2017; Vernooij et al., 2009)).

#### Fatigue

The symptom scale ‘fatigue’ of the European Organisation for Research and Treatment of Cancer Quality of Life Questionnaire (EORTC QLQ-C30) was calculated, ranging from 0–100, with higher scores representing a higher symptom burden (Aaronson et al., 1993). A score of ≥ 39 indicated high fatigue (Giesinger et al., 2020).

#### Physical fitness

Physical fitness was measured with a maximal cycle CPET using a ramp protocol, including continuous breathing gas analysis and electrocardiogram monitoring. Relative maximum oxygen uptake (VO_2peak_) was calculated as an average over the final 30 s of exercise divided by body weight at baseline.

### Statistical analyses

As this paper does not analyze the primary outcomes of the PAM study, all results are exploratory.

Between-group differences in demographic and clinical characteristics were analyzed using Independent Samples T-Tests for continuous variables and Chi-Square tests for categorical variables.

#### Age and white matter integrity

Mean FA and mean MD values were extracted as measures for whole brain white matter microstructural integrity (overall FA and overall MD). To verify the expected association with age, we performed regression analyses between age and overall FA and MD at baseline.

#### Intervention effects on whole brain and regional white matter integrity

For the primary analyses, the intention-to-treat principle was applied and based on a complete case data set. To assess between-group differences on whole brain white matter integrity, we used multiple regression analyses with overall FA or MD at follow-up as outcome, and randomization, baseline measurement, and stratification factors as covariates.

To assess regional effects, we performed voxel-wise analyses to identify differences in white matter integrity between the exercise and control group. Difference maps (FA and MD) were calculated by subtracting baseline from follow-up measurements. A nonparametric general linear model with 5000 permutations, for both DTI metrics, was applied using the *randomize* tool (Winkler et al., 2014) of FSL (Smith et al., 2004). Threshold-free cluster-enhancement (TFCE) was used to correct for multiple comparisons (Smith & Nichols, 2009). Clusters were considered statistically significant with family-wise error (FWE) corrected threshold of 0.05.

#### Association between DTI metrics and cognition

For significant clusters of changes in DTI metrics, we investigated whether changes in white matter integrity were associated with changes in cognitive functioning (HVLT-R total recall and ACS Reaction Time); we extracted mean FA/MD values from the significant clusters and performed multiple regression analyses with the difference scores including baseline (HVLT-R total recall or ACS Reaction Time) as covariate. These analyses are intended to provide a broader perspective and to explore whether the DTI results align as an underlying mechanism.

#### Per protocol analyses and highly fatigued patients

We repeated the above mentioned (between-group) DTI analyses for patients who reached at least 80% adherence to the intervention program ((number of attended sessions / number of offered sessions) *100) and, because of previous findings, for highly fatigued patients at baseline (EORTC QLQ-C30 ‘fatigue’ ≥ 39) (Koevoets et al., 2022).

All statistical analyses were executed with SPSS version 26.0 (IBM Corp, 2019) and the critical two-sided alpha value was set at 0.05.

## Results

### Patient characteristics

We initially included 181 patients. For this study, we included patients with baseline and follow-up MRI data (*n* = 142). Most frequent reason for drop-out is missing MRI data due to the COVID-19 pandemic, see Fig. 1. Drop-outs had a lower age at menopause (dropouts: 44.7 ± 5.8 yrs. vs inclusions: 47.9 ± 5.8 yrs.), more often received a combination of neoadjuvant and adjuvant chemotherapy and were included closer to chemotherapy completion (dropouts: 2.4 ± 0.6 yrs. vs inclusions: 2.7 ± 0.6 yrs.). No other differences were observed. This resulted in a final dataset of 69 patients in the intervention group (mean age = 52.3 ± 8.9yrs) and 72 patients in the control group (mean age = 53.2 ± 8.6yrs). Groups were comparable on all demographic and clinical characteristics, except for a marginally significant difference in psychotropic medication use (Table 1). In the intervention group, an attendance of ≥ 80% to the intervention program was reached by 51 patients (74%), with a median attendance of 88% (range 0–100%, mean = 81% ± 21).

**Table 1:** Baseline demographic and treatment characteristics

	Intervention group *(n* = *69)*	Control group *(n* = *72)*	*P*
Age (yrs.)	52.3 (8.9)	53.2 (8.6)	0.545
Education level (*n* (%))			
High	33 (47.8)	30 (41.7)	
Middle	36 (52.2)	42 (58.3)	
Low	0 (0)	0 (0)	0.501
Physical fitness (VO_2peak_ in ml/min/kg)	23.6 (4.6)	24.8 (6.1)	0.162
Menopausal status (*n* (%))			
Pre/peri	9 (13.0)	7 (9.7)	
Post	60 (87.0)	65 (90.3)	0.602
Age of menopause (yrs.)	47.9 (6.4)	47.7 (5.4)	0.808
Time since diagnosis (yrs.)*	3.1 (0.7)	3.1 (0.6)	0.860
Tumor grade (*n* (%))			
I	10 (14.5)	5 (6.9)	
II	28 (40.6)	31 (43.1)	
III	24 (34.8)	28 (38.9)	
Unknown	7 (10.1)	8 (11.1)	0.532
Surgery (*n* (%))	69 (100)	72 (100)	-
Chemotherapy timing (*n* (%))			
Neoadjuvant	31 (44.9)	34 (47.2)	
Adjuvant	36 (52.2)	36 (50.0)	
Both	1 (1.45)	1 (1.4)	
Unknown	1 (1.45)	1 (1.4)	0.995
Time since completion of chemotherapy (yrs.)*	2.7 (0.7)	2.7 (0.6)	0.553
Radiotherapy (*n* (%))			
Yes	53 (76.8)	55 (76.4)	
No	16 (23.2)	17 (23.6)	0.953
Targeted therapy (*n* (%))			
Yes	16 (23.2)	15 (20.8)	
No	53 (76.8)	56 (77.8)	
Unknown		1 (1.4)	0.591
Endocrine therapy (*n* (%))			
Yes	43 (62.3)	45 (62.5)	
No	26 (37.7)	27 (37.5)	0.982
Medication use (*n* (%))			
Cardiovascular	14 (20.3)	14 (19.4)	0.900
Anti-diabetic	1 (1.4)	1 (1.4)	0.976
Psychotropic	21 (30.4)	12 (16.7)	0.054
Pain medication	11 (15.9)	12 (16.7)	0.907

Values indicate mean (SD), unless indicated otherwise. P values indicate overall group differences

^*^For time since diagnosis, average years were based on 62 intervention patients and 67 control patients. For time since completion of chemotherapy, average years were based on 64 intervention patients and 62 control patients

### Age and white matter integrity

At baseline, older age was associated with a lower overall FA (R = −0.43, B = −0.0006, 95%CI: −0.0009; −0.0004) and a higher overall MD*1000 (R = 0.59, B = 0.0020, 95%CI: 0.015; 0.0025).

### Intervention effects on whole brain and regional white matter integrity

Overall FA (B = 0.0001, 95%CI: −0.0013; 0.0011) and overall MD*1000 (B = 0.0003, 95%CI: −0.0021; 0.0027) did not differentially change in the intervention group compared to the control group over the 6-month follow-up period (Supplementary Table 3). The change scores from baseline to follow-up in FA and MD clusters were not different for the two groups, either.

### Per protocol analyses and highly fatigued patients

Results of the per protocol analysis did not differ from the intention-to treat analyses.

In highly fatigued patients, no significant difference in the intervention group (*n* = 32) compared to the control group (*n* = 24) for both overall FA (B = −0.0011, 95%CI: −0.0033; 0.0011) and overall MD*1000 (B = 0.0009, 95%CI: −0.0027; 0.0045) was detected.

**Fig. 2:**
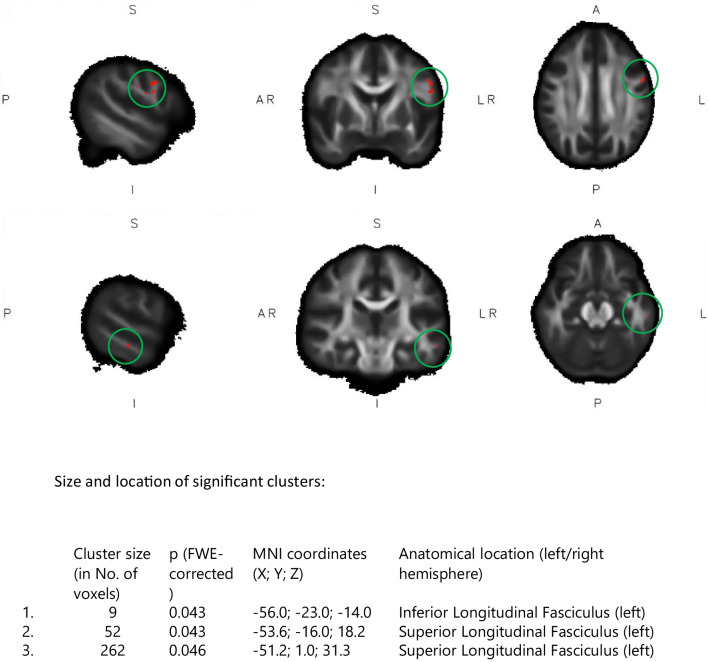
Significant clusters for fractional anisotropy decline for highly fatigued patients in the intervention compared to the control group

Voxel-wise analyses revealed a decrease in FA in the left inferior longitudinal fasciculus (one cluster, lower panel) and left superior longitudinal fasciculus (two clusters, upper panel) for the highly fatigued patients in the intervention group compared to those in the control group (see Fig. 2).

Mean FA from the significant between-group clusters was not predictive of HVLT-R total recall (B = −30.5, 95%CI: −128.8; 67.7) or ACS Reaction Time performance (B = 385, 95%CI: −1280; 2050).

## Discussion

We investigated the effects of a 6-month exercise program on white matter integrity, 2–4 years after breast cancer diagnosis in patients with affected cognitive functioning. We observed no significant exercise effects on white matter integrity assessed by the DTI metrics FA and MD. Also, the per-protocol analysis of the adherent participants did not show an intervention effect. In highly fatigued patients, we observed a decline in FA in the left inferior and superior longitudinal fasciculus in the intervention group compared to the control group. The direction of this result was unexpected.

Our observation of no significant exercise effects on white matter integrity in breast cancer patients might be explained by the lower mean age of participants in our study compared to the general breast cancer population. Previous research suggests that younger patients may report more cognitive complaints even when objective deficits are not present or when those deficits are mild (Hanson et al., 2020). Another explanation might be that there is limited white matter damage in breast cancer patients since the long-term effects of chemotherapy on white matter integrity are inconclusive. Long-term effects have been described after high-dose but not after normal-dose chemotherapy (Billiet et al., 2018; Koppelmans et al., 2014; Stouten-Kemperman et al., 2015). Additionally, a longitudinal study reported that at 3–4 years post-treatment, FA values returned to baseline in chemotherapy-treated patients (Billiet et al., 2018). This indicates that white matter integrity might have recovered over time, even in patients with long-lasting cognitive complaints, as in the current study.

From exploratory analyses in highly fatigued patients at baseline, we previously reported significant cognitive improvement, no effects on brain perfusion, and an unexpected decrease in hippocampal volume post-exercise, which was also related to improved memory functioning (Koevoets et al., 2023a, 2023b, 2022b). In the current study, we found in this group a decrease in FA in the left inferior and superior longitudinal fasciculus. Similar to the hippocampal volume decrease after exercise, the direction of this finding was unexpected. However, comparable results have been reported previously. A single-arm exercise intervention study found a decrease in FA in an aging population (Clark et al., 2019), but the effect was attributed to normal aging. Additionally, in a randomized controlled trial in older adults at risk for dementia (*n* = 60), Stephen et al. observed that a multidomain lifestyle intervention resulted in a decrease in FA, which was related to an improvement in a composite score of cognitive functioning (Stephen et al., 2020). They suggested that the decrease in FA was not related to demyelination, but rather a different pathophysiological change.

Potential limitations should be mentioned. First, we limited our analyses to two cognitive tests to reduce the risk of statistical type I errors, but we might, therefore, have missed a significant relation between decreased FA and cognitive improvement. Second limitation is the lower mean age of participants in our study compared to the general breast cancer population. Third, due to the COVID-19 pandemic, follow-up data of 19 patients were missing. Nevertheless, despite suboptimal follow-up, this is a large trial including brain MRI, which should be considered a major strength of the study. Additionally, the novel pipeline with state-of-the-art registration techniques which avoided the skeletonization step used in conventional TBSS increased the sensitivity of our DTI analyses. We further included breast cancer patients who were relatively inactive and were cognitively affected at baseline, leaving room for improvement. Also, patients underwent a partly supervised exercise intervention of high intensity, which was partly supervised with both aerobic and strength training (four hours/week for six months).

## Conclusions

A 6-month exercise program of moderate-to-high intensity did not affect white matter integrity in chemotherapy-treated breast cancer patients with cognitive problems. However, in a group of highly fatigued patients, a decrease in FA was observed after the intervention. The direction of this effect was unexpected. Mechanistic explanations of exercise effects in highly fatigued breast cancer patients with cognitive problems need further elaboration.

## Supplementary Information

Below is the link to the electronic supplementary material.

Supplementary file1 (DOCX 19 KB)

## Data Availability

The datasets generated during and analyzed during the current study are available from the corresponding author on reasonable request.

## Declarations

The study was approved by the Medical Ethics Committee of the UMCU and all patients provided written informed consent before baseline measurements.
