# Supplementary material for: Effect of physical exercise on white matter microstructure in chemotherapy-treated breast cancer patients: a randomized controlled trial (PAM study)
Source: Brain Imaging Behav. 2025 Jan 13;19(2):291–301. doi: 10.1007/s11682-024-00965-9 (PMC11978695; doi:10.1007/s11682-024-00965-9)
Supplement: Supplementary file 1 — Supplementary file1 (DOCX 19 KB) [file 11682_2024_965_MOESM1_ESM.docx]

**Supplementary material**

**Supplementary table 1.** Content of the Amsterdam Cognition Scan.

| **Test domain** | **Online test** | **Main outcome measures** | **Traditional equivalent** |
| --- | --- | --- | --- |
| **Learning and memory** | Wordlist Learning  Wordlist Delayed Recall  Wordlist Recognition | Total number of correct responses (Learning: trials 1 to 5) | Dutch version of Rey Auditory Verbal Learning Test (immediate recall, delayed recall, and recognition) |
| **Attention and working memory** | Box Tapping  Digit Sequences I  Digit Sequences II | Total number of correctly repeated sequences | Corsi Block-Tapping Test  WAIS-III Digit Span forward  WAIS-III Digit Span backward |
| **Processing speed** | Reaction Time  Connecting the Dots I | Mean reaction time (msec)  Completion time (sec) | Visual Reaction Time (subtest FePsy)  Trail Making Test A |
| **Executive functioning** | Connecting the Dots II  Place the Beads | Completion time (sec)  Total number of extra moves | Trail Making Test B  Tower of London, Drexel University (ToL-dx) |
| **Motor functioning** | Fill the Grid | Completion time (sec) | Grooved Pegboard |

**Supplementary table 2.** Supervised exercise program of the PAM study.

| **Week** | **Aerobic** | **Strength** |
| --- | --- | --- |
| **1-4** | 40%-60% HRR | One circuit of 20-25 repetitions. Weights based on 20-RM tests (repeated every 4 weeks).  Exercises: legs (squat, lunges, calve raises), arms (biceps curl, triceps extension), shoulder (shoulder press), thorax (Barbell bench press), back (rowing). Abdomen: crunch 30-40 repetitions. |
| **5-9** | 60%-70% HRR 15-20 min,  plus 70%-89% HRR 5-10 min |  |
| **10-17** | Interval training: 10x30 sec vigorous to maximal exercise, alternated with 1 min active rest,  plus 10 min 60%-75% endurance | Two circuits of 15-20 repetitions. Weights based on 15-RM tests (repeated every 4 weeks).  Exercises: legs (squat), arms (biceps curl, triceps extension), shoulder (shoulder press), thorax (Barbell bench press), back (rowing). Abdomen: crunch 30-40 repetitions; hoover/planking 2x 45 sec. |
| **18-26** | Interval training: 2 circuits of 8x30 sec vigorous to maximal exercise, alternated with 1 min active rest,  plus 5 min 60%-75% endurance |  |

Abbreviations: PAM, Physical Activity and Memory; HRR, heart rate reserve; RM, repetition maximum.

Supplementary material (text): Description of the adjusted TBSS pipeline

In this modified pipeline B0 volumes were extracted from the DTI data and skull stripped using FSL’s Brain Extraction Tool. Then, DTI images were corrected for patient motion and residual eddy current distortion using eddy, and a diffusion tensor model was fitted at each voxel using FSL’s dtifit program. This generated individual FA and MD maps. The bright halo of voxels from the eddy current distortions surrounding the individual FA maps was removed using the first step of TBSS. The ANTs registration tool was used for rigid, affine, and non-linear (SyN) registration of each FA map to its corresponding individual skull-stripped T1-weighted image. This generated FA-to-T1 images. A group-wise T1 template was generated based on the individual skull-stripped T1-weighted scans of both the baseline and follow-up measurements. The warping parameters of individual T1-weighted images were used to register the individual FA-to-T1 images to the common space of the group-wise T1 template. This generated FA-to-Template images. The FA-to-Template images were smoothed with a 6mm full-width at half-maximum (FWHM) Gaussian kernel. A binary mask was constructed based on the mean of all FA-to-Template images to analyze voxels with a mean FA value of > 0.2, restricting the analyses mainly to white matter.

Warping parameters generated from creating FA-to-T1 images and FA-to-Template images were then used to also register individual MD images to the common space of the group-wise T1 template, which resulted in MD-to-Template images. Similar to the FA images, MD images were smoothed with a 6mm FWHM Gaussian kernel.

**Supplementary table 3.** Overall FA and MD values at baseline and follow-up, separately for the intervention and control group.

|  | **Intervention group *(n=69)*** | **Control group (n=72)** | **Treatment effect† (95% CI)** |
| --- | --- | --- | --- |
| **Fractional Anisotropy (FA)**  **Baseline**  **Follow-up** | 0.324 (0.013)  0.323 (0.013) | 0.323 (0.013)  0.322 (0.013) | 0.0001 (-0.0013; 0.0011) |
| **Mean Diffusivity* (MD)**  **Baseline**  **Follow-up** | 0.794 (0.031)  0.794 (0.029) | 0.796 (0.029)  0.796 (0.031) | 0.0003 (-0.0021; 0.0027) |

Values indicate mean (SD).

*x 10^-3^ mm^2^/s.

† The intervention effect is the regression coefficient of a linear regression analysis adjusted for baseline, age, and endocrine therapy.
